# Effects of individual characteristics and local body functions on sweating response: A review

**DOI:** 10.1007/s00484-024-02758-7

**Published:** 2024-08-14

**Authors:** Zhuoxi Niu, Tomonobu Goto

**Affiliations:** Department of Architecture and Building Science, Tohoku University, Sendai, Japan

**Keywords:** Sweat, Individual characteristic, Local body function, Thermoregulation

## Abstract

In this study, we conducted a literature review to deepen our understanding of the sweating response of the thermoregulatory system, focusing on the influence of individual characteristics and local body functions. Among the factors related to individual characteristics, improvement in aerobic fitness had a positive effect on the sweating response, whereas aging exerted an inhibitory effect. Short-term artificial acclimation and seasonal heat acclimatization promoted sweating, whereas long-term geographical acclimatization suppressed sweating. Male exhibited higher sweat rates than female when the metabolic heat production was high. Individuals with smaller surface area-to-mass ratios tended to have higher sweat rates than those with larger ratios. Regarding local body functions, sweat distribution in the resting state showed high regional sweat rates in the lower limbs and torso, with higher values in the lower limbs when in the supine position and higher values in the torso when in the seated position. During exercise, the regional sweat rates was high in the torso, whereas the limbs exhibited relatively low sweat rates. These differences in sweat distribution stem from the thermoregulatory potential of each body region, which aims to efficiently regulate body temperature. Local effects have only been examined in the thigh and forearm, with temperature coefficient Q_10_ ranging from 2 to 5. Only the forehead showed significantly high thermosensitivity among all body regions.

**Supplementary Information:**

The online version contains supplementary material available at 10.1007/s00484-024-02758-7.

## Introduction

Humans are homeothermic animals that maintain their body temperature within a certain range by exchanging heat with the environment to ensure optimal physiological functioning (Nielsen 1969; Romanovsky 2018). The heat exchange between the human body and environment can be described by the following heat balance equation:

1 $$S=M-W-C-R-E-{C}_{res}-{E}_{res}$$

where S is the heat storage [W.m^−2^]; M is the metabolic rate [W.m^−2^]; W is the absolute external work rate [W.m^−2^]; C and R represent sensible heat loss via convection and radiation from the skin surface [W.m^−2^]; E represents latent heat loss via evaporation [W.m^−2^]; C_res_ and E_res_ are sensible and latent heat loss by respiration [W.m^−2^], respectively.

When environmental temperature rises, C and R decrease correspondingly, resulting in an increase in S. During exercise, (M—W) increases correspondingly, also resulting in an increase in S. As a thermoregulatory function, the sweating response is induced to increase E, thereby suppressing the increase in S. Here, not all sweat can evaporate; for example, sweat cannot evaporate beyond the maximum evaporation rate, known as E_max_. Nevertheless, sweating plays a crucial role in suppressing the increase in S, that is, in preventing the rise in body temperature.

Despite the primary factors of sweating response are a combination of the thermal environment and metabolic heat production (M—W) (Gagnon et al. 2013b), sweating responses vary among individuals even under identical environmental conditions and the same amount of physical activity, indicating inter-individual differences. Furthermore, sweating responses elicited in different body regions are not uniform within the same individual, highlighting intra-individual differences. Inter-individual differences in sweating responses are influenced by individual physiological characteristics, such as aerobic fitness, age, heat acclimation, sex, and body surface area-to-mass ratio (Havenith and van Middendorp 1990; Havenith et al. 1995). These individual characteristics can be regarded as the outcomes of general or systemic adaptations to thermoregulation. In contrast, intra-individual differences across various body regions are influenced by local body functions inherent in each body region, such as sweat distribution and local effect, which refers to the extent of the increased sweating response of each body region to systemic body temperature elevation (Kuno 1934) and the extent of the increased sweating response of a specific body area to local skin temperature elevation (van Beaumont and Bullard 1965), respectively. These local functions can be considered the outcomes of localized adaptations related to thermoregulation. Furthermore, although it does not influence the differences in sweating responses across various body regions, thermosensitivity (Hardy and Oppel 1937; Hensel 1981) can also be considered a type of local body function. Differences in thermosensitivity affect the extent of systemic sweating response to local skin temperature changes in the respective body regions.

It is crucial to elucidate the influence of individual characteristics and local body functions to deepen our understanding of the complex physiological process of sweating response and how it contributes to thermoregulation. Therefore, this study aimed to conduct a review on the individual characteristics and local body functions involved in sweating responses.

Regarding individual characteristics, previous research has provided somewhat consistent insights; however, it is not realistic to discuss their effects on sweat responses quantitatively. Thus, this study aimed to organize the qualitative insights advocated in previous studies. Additionally, the findings on these individual characteristics can also be utilized to better understand experimental data concerning local body functions, which will be discussed later in this article. Concerning local body functions, there is no consistent consensus in the existing literature; however, it is possible to discuss their effects quantitatively based on the experimental data obtained from these studies. We aimed to organize and compare previous experimental data to derive comprehensive insights. Specifically, this research reviewed studies that measured regional sweat rates under uniform thermal conditions, and the experimental data were organized and compared for two conditions: resting and exercise. Furthermore, experimental data on local effect and thermosensitivity were organized by reviewing studies that measured sweat rates under nonuniform thermal conditions by applying local thermal stimuli to specific body regions.

## Effects of individual characteristics on sweat response

### Methods

Authors conducted a literature search to investigate the impact of individual physiological characteristics on the sweating response. Databases such as PubMed, Scopus, Web of Science, and J-STAGE were used from the earliest to December 2023. The search terms included keywords related to sweating response combined with keywords for various individual characteristics. The specific search terms are summarized in Appendix 1. The reference lists of the identified articles were manually checked. Relevant studies were also sourced from bibliographies of review articles. Titles and abstracts were screened for inclusion. When the abstract information was ambiguous, the full text was checked. Studies were assessed against the following criteria: (1) sweat rate measurement via subjective experiments, (2) evaluation of the effects of individual characteristics, and (3) publication of original research articles in English or Japanese.

### Results

#### Age

A decline in the defense mechanisms against heat stress has been observed due to aging, resulting in reduced sweat production (Shoenfeld et al. 1978; Kenney and Anderson 1988; Inoue et al. 1995, 1998; Coull et al. 2021). This reduction may not uniformly affect the entire body but may gradually spread from the lower limbs to the torso (Inoue 1996; Inoue and Shibasaki 1996). Some researchers have concluded that the reduction in sweat production is caused by a decrease in the number of sweat glands (Mackinnon 1954; Siver et al. 1964). Other studies have demonstrated that this decrease is attributable to a decline in sweat gland output (Anderson and Kenney 1987; Kenney and Fowler 1988; Inoue et al. 1991; Smith et al. 2013), which is presumed to result from reduced cholinergic sensitivity (Inoue et al. 1999a, 1999b). In addition, it has been speculated that the body temperature threshold for sweating onset may increase in older individuals (Hellon and Lind 1956; Foster et al. 1976). Furthermore, a decrease in the density of thermoreceptors (Chang et al. 2004; Goransson et al. 2004; Panoutsopoulou et al. 2009) and transmission speed of nerve signals (Dorfman and Bosley 1979; Bouche et al. 1993; Rivner et al. 2001) have been confirmed, suggesting that these factors may also influence the reduction in sweat production.

#### Heat acclimation/acclimatization

Exposure to hot environments leads to two types of adaptations that affect sweating responses: heat acclimation induced by specific experimental conditions, such as artificial climate chambers, and heat acclimatization occurring naturally owing to seasonal or geographical conditions.

Heat acclimation usually lasts for 1–2 weeks in environments with temperatures ranging from 30 to 40 °C, and a significant enhancement in sweating responses is widely observed (Wyndham et al. 1964; Peter and Wyndham 1966; Nielsen et al. 1993, 1997; Poirier et al. 2015, 2016). One hypothesis regarding the mechanism of this phenomenon suggests that short-term heat acclimation lowers the body temperature threshold, which triggers sweating responses (Fox et al. 1963; Nadel et al. 1974; Roberts et al. 1977; Shvartz et al. 1979; Hessemer et al. 1986; Cotter et al. 1997; Buono et al. 1998). There is also a possibility that cholinergic sensitivity to body temperature increases (Henane and Bittel 1975; Libert et al. 1983). Moreover, it was discovered that the increase in the regional sweat rate on the limbs was higher than that on the torso. This suggests a redistribution of sweat to effectively utilize the limbs, which have a higher evaporative capacity (Hofler 1968; Shvartz et al. 1979; Regan et al. 1996; Smith and Havenith 2019). However, counterarguments suggest that this phenomenon is simply a result of body regions with inherently lower sweat rates experiencing increased sweat production owing to short-term heat acclimation (Patterson et al. 2004).

Natural heat acclimatization can be categorized as seasonal heat acclimatization, wherein the body adapts to outdoor environmental exposure during specific seasons, and geographical heat acclimatization, wherein the body adapts to the environmental conditions of a particular region by residing there. Research on seasonal heat acclimatization has primarily focused on acclimatization to hot summer temperatures. It has been found that summer seasonal heat acclimatization increases sweat production (Inoue et al. 1995; Bates and Miller 2008; Lee et al. 2015; Notley et al. 2020). A decrease in body temperature threshold has also been observed (Torii et al. 1985; Torii and Nakayama 1993; Lui et al. 2014). In contrast, an increase in cholinergic sensitivity to body temperature has also been observed (Taniguchi et al. 2011; Lei et al. 2021). In examining the effects of geographical acclimatization, a comparison of sweat responses between indigenous populations of non-tropical and tropical climates revealed that individuals from tropical climates exhibited lower sweat rates and delayed sweating onset (Hori et al. 1976; Matsumoto et al. 1993; Lee et al. 1998, 2004, 2007, 2009). Upon relocating to temperate regions for several years, individuals from tropical climates exhibited a reduction in the threshold of sweating onset and an increase in sweat production (Hori et al. 1979; Saat et al. 1999; Lee et al. 2002; Wijayanto et al. 2012). Some researchers have speculated that the basal metabolic rate of individuals in tropical climates decreases owing to geographical acclimatization (Lee et al. 2013; Lee and Kim 2018). Others have concluded that individuals from tropical climates reduce their reliance on sweating responses by engaging in more vigorous cutaneous vascular activities (Lee et al. 2011; Wakabayashi et al. 2011; Wijayanto et al. 2011).

#### Sex

To investigate sex differences, researchers have induced sweating in participants through various experimental procedures, such as passive heating (Herrmann et al. 1952; Fox et al. 1969; Bittel and Henane 1975; Inoue et al. 2005), and exercise under a certain intensity (Wyndham et al. 1965; Morimoto et al. 1967; Weinman et al. 1967; Paolone et al. 1978; Avellini et al. 1980; Frye and Kamon 1981; Horstman and Christensen 1982; Keatisuwan et al. 1996; Ichinose-Kuwahara et al. 2010). These studies have shown that females generally produce less sweat, leading to the widespread acknowledgment of this as a sex difference.

Nevertheless, physical characteristics such as body size and aerobic fitness levels, should be considered (Havenith 2001, Havenith et al. 2008). For instance, Schwiening et al. (2011) claimed that the sex difference in sweat rate under the same relative exercise intensity (%VO_2 max_) reported by Ichinose-Kuwahara et al. (2010) could be explained by the sex difference in the absolute external work rate. Gagnon et al. (2008) pointed out that sex difference in sweat rate is attributable to differences in metabolic heat production; therefore, sex difference is negligible when metabolic heat production is unchanged. When metabolic heat production increases to a certain extent, males tend to sweat more than females, even under the same metabolic heat production (Gagnon and Kenny 2011, 2012).

The influence of female hormone secretion during different menstrual cycles was considered. During the luteal phase, progesterone tends to increase body temperature, whereas during the follicular phase, estrogen tends to decrease body temperature. Therefore, the hormonal effects on body temperature may have corresponding effects on sweat responses by inducing both promotion and inhibition (Stephenson and Kolka 1993; Charkoudian and Stachenfeld 2016).

Typically, no difference is observed in the total number of active sweat glands between females and males (Szabo 1967; Bar-Or et al. 1968; Knip 1969). Therefore, the variance in sweat response is not attributed to the number of sweat glands but rather to differences in sweat gland output. Higher cholinergic sensitivity in sweat glands has been observed in males than in females (Gagnon et al. 2013a). Another difference is the sweat distribution. Although both males and females exhibit similar patterns, males tend to distribute sweat more from the torso, whereas females sweat more from the arms, hands, and legs (Havenith et al. 2008; Smith and Havenith. 2011, 2012).

#### Body surface area-to-mass ratio

Experiments have suggested that females exhibit greater tolerance to higher temperatures under high-humidity conditions than males, while males demonstrate greater tolerance to high temperatures under low-humidity conditions (Shapiro et al. 1980; Frye and Kamon 1983). Notley et al. (2017) argued that such results are heavily influenced by morphological characteristics, specifically body surface area-to-mass ratio. Typically, female have smaller body sizes than male, resulting in higher body surface area-to-mass ratios. This morphological characteristic facilitates sensible heat loss through cutaneous vasodilation, enabling individuals to endure prolonged periods in hot and humid environments. In contrast, individuals with larger body sizes have a smaller body surface area-to-mass ratio, leading to a greater reliance on the sweating response than individuals with smaller body sizes.

From an anthropological perspective, according to Bergmann’s rule, homeothermic animals tend to have larger body sizes in colder regions and smaller body sizes in warmer regions (Bergmann 1847). Allen’s rule states that animals inhabiting colder regions tend to have shorter limbs and bodies than those inhabiting warmer regions (Allen 1877). Indeed, a significant negative correlation was observed between the body weight of indigenous populations in various regions and average annual temperature. Specifically, the body surface area-to-mass ratio of tropical indigenous populations was found to be higher than that of indigenous populations in other regions (Roberts 1953, 1978; Katzmarzyk and Leonard 1998), suggesting a superior sensible heat loss capacity (Hori and Ihzuka 1986). Minimizing latent heat loss through sweating and relying on sensible heat loss remains crucial in hot and humid tropical climates. To adapt to this type of environment, there is a reduction in sweat production and changes in body morphology.

#### Aerobic fitness

Researchers typically used two groups with different maximal oxygen consumption (VO_2 max_) exercises at a fixed relative exercise intensity (%VO_2 max_) and found that participants with high aerobic fitness levels had higher sweat rates (Davies 1979; Gass et al. 1991; Ho et al. 1997; Fritzsche and Coyle 2000; Mora-Rodriguez et al. 2010; Ichinose-Kuwahara et al. 2010). Yet, as mentioned in the previous section, metabolic heat production plays a crucial role in sweating response (Gagnon et al. 2008). The differences in sweat rate were primarily due to differences in metabolic heat production rather than VO_2 max_. (Jay et al. 2011; Cramer et al. 2012).

On the other hand, pilocarpine iontophoresis, which acts directly on sweat gland receptors independent of the sympathetic system, is frequently employed by researchers to investigate sweat rate (Madeira et al. 2010; Inoue et al. 2014; Lee et al. 2014). Because this method reflects the capacity of the sweat gland itself, aerobic fitness has been suggested to improve the sweating response by increasing cholinergic sensitivity to changes in body temperature (Buono and Sjoholm 1988; Buono et al. 1992; Wilson et al. 2010). Okazaki et al. (2002) argued that this enhancement can be achieved by adjusting the body temperature threshold.

## Effects of local body functions on sweat response

### Methods

A literature search on the local body functions of (1) sweat distribution, (2) local effect and (3) thermosensitivity were conducted using the PubMed, Scopus, Web of Science and J-STAGE databases from their inception to December 2023. Different search terms and selection criteria were used for (1) – (3) respectively. The search process and flow charts are presented in Appendix 1.

Regarding sweat distribution, due to the aim is to investigate the contribution of each body part to thermoregulation through sweating, the included studies had to ensure the use of adequate measurement sites to approximate sweat distribution across the body. Therefore, studies focusing on only a few body parts were excluded.

### Results

#### Sweat distribution during resting state

Five articles were selected for a detailed review. The experimental information collected from those articles were listed in Table 1. It should be noted that Kuno (1956) measured the accumulated regional sweat rate during a period from before heat exposure until sometime after, while measurements in other studies were taken only after heat exposure.

**Table 1:** Experimental information from previous studies conducted under resting state

Author	Air temp	Participants information	Position	Measuring method	Measurement sites (number of sites within the body part)
Hertzman et al. 1952	24–38 ℃	22 young males (age: unknown)	Supine	Ventilated capsule	forehead, cheek, upper arm, lower arm, chest, abdomen, thigh, leg, palm, sole
Park and Tamura 1992a	25, 28, 31, 34, 37 ℃	10 females (age: 24 ± 4 years)	Supine	Evaporimeter	forehead, cheek, neck (2), shoulder, chest (2), abdomen (3), upper back (3), lower back, axilla, upper arm (2), lower arm (2), palm, dorsal hand, inguinalis, buttock, thigh (2), leg (2), sole, dorsal foot
Chung and Tamura 1998	28, 34, 37 ℃	10 females (age: 23 ± 2 years)	Supine, seated	Evaporimeter	forehead, chest, abdomen (2), upper back, lower back, upper arm, lower arm, palm, dorsal hand, thigh, leg (2), dorsal foot
Coull et al. 2021	32 ℃	14 young males (age: 24 ± 2 years), 14 old males (age: 68 ± 5 years)	Seated	Sweat pad	shoulder, chest (2), abdomen (4), upper back (2), lower back (4), upper arm (2), lower arm (2), palm, dorsal hand, thigh (4), leg (3), ankle (2), sole, dorsal foot
Kuno 1956	39–40 ℃	4 males and 4 females (age: unknown)	Standing	Round dish	forehead, cheek, neck, chest, abdomen, upper back, lower back, axilla (2), upper arm (2), lower arm, palm, dorsal hand, buttock, thigh (2), leg, sole, dorsal foot

**Fig. 1:**
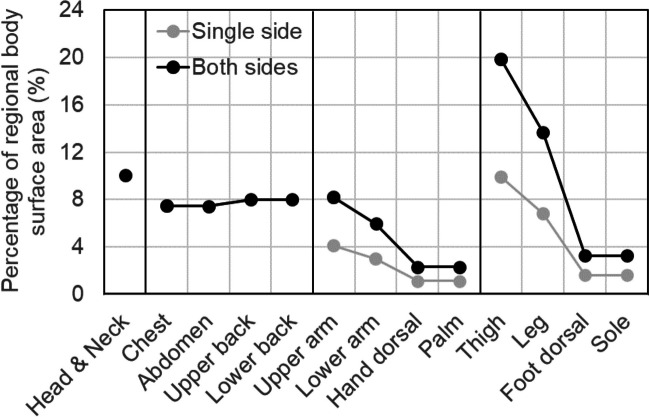
Percentage of regional body surface area

To facilitate the comparison of the regional sweat rates, data from 13 regions were considered: forehead, chest, abdomen, upper back, lower back, upper arm, lower arm, dorsal hand, palm, thigh, leg, dorsal foot, and sole. Data on the cheek, neck, shoulder, axilla, inguinal region, buttock, and ankle were excluded. In some studies, the 13 specific regions were not measured. The regional sweat rate was expressed per unit area. However, considering their contribution to overall body temperature regulation, it is important to consider the skin surface area of each body region. The percentage of regional body surface area is provided in Fig. 1 for reference. These values were calculated based on data from Yu et al. (2010). The grey lines represent the proportion of one side of the limbs, whereas the black lines indicate a combined proportion of both sides.

**Fig. 2:**
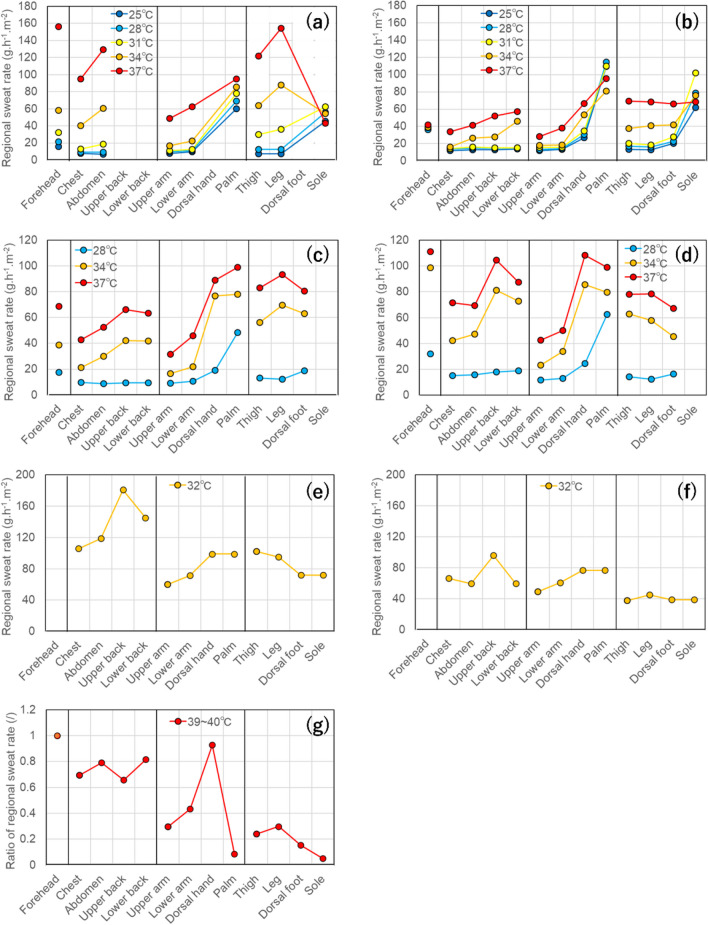
Regional sweat rate from previous studies conducted under resting state. **a:** data of supine participants collected by Hertzman et al. (1952), **b:** data of supine participants collected by Park and Tamura (1992a), **c:** data of supine participants collected by Chung and Tamura (1998), **d:** data of seated participants collected by Chung and Tamura (1998), **e**: data of seated young participants collected by Coull et al. (2021), **f:** data of seated old participants collected by Coull et al. (2021),**g**: data of standing participants collected by Kuno (1956)

The regional sweat rates during the resting state, as measured in the studies listed in Table 1, are shown in Fig. 2. In cases where measurements were conducted at multiple locations for a single region, the regional sweat rate [g.h^−1^. m^−2^] was calculated as follows:

2 $$RSR=\frac{{SR}_{1}\times {A}_{1}+{SR}_{2}\times {A}_{2}+\dots +{SR}_{n}\times {A}_{n}}{{A}_{1}+{A}_{2}+\dots +{A}_{n}}$$

where SR is the sweat rate of each segmented skin area belonging to a body region [g.h^−1^. m^−2^] and A is the area of each segmented skin area [m^2^]. If the segmented area is unknown, a simple arithmetic mean is used.

Regarding the measurement by Kuno (1956), as shown in Fig. 2g, the measurement time and regional skin area were not provided. Therefore, instead of using the unit g.h^−1^. m^−2^, the data are presented as the ratio of the maximum sweat rate for each region.

Owing to differences observed in sweat distribution based on the participants’ postures, this research will describe the data separately for the supine, seated, and standing positions.

Figure 2a–c show the data collected in the supine position. The data in Fig. 2a represent the aggregation of 61 experimental results conducted at various air temperatures ranging from 24 °C to 38 °C, categorized into five temperature conditions corresponding to the data in Fig. 2b. When comparing the data from each study, it was evident that under similar temperature conditions, the sweat rate in Fig. 2a was higher than that in the other two studies. This discrepancy can be attributed to the fact that Fig. 2a was measured using ventilated capsules, whereas Fig. 2b and c were measured using an evaporimeter. Park and Tamura (1992b) demonstrated that sweat rate measured using ventilated capsules exceeded that measured using an evaporimeter. Yamada and Tamura (2012a, 2012b) noted that there was a phenomenon known as hidromeiosis, in which sweat secretion is inhibited by skin moisture during natural sweating. This phenomenon did not occur in the ventilated capsules because of the constant airflow circulating inside, which facilitated the immediate evaporation of sweat. Therefore, doubt remains regarding the accuracy of the absolute values in Fig. 2a. Nevertheless, assuming that the relative relationship between the regional sweat rates of different body regions remained consistent with natural sweating conditions, we proceeded by comparing the data.

A common feature across all Fig. 2a to c was that when the air temperature was below 28 °C, regional sweat rates of the torso (chest, abdomen, upper back, lower back), upper limbs (upper arm, lower arm), and lower limbs (thigh, leg) were low, with minimal differences between the regions. Under conditions where the air temperature ranged from above 28 °C to 34 °C, a relatively significant increase occurred in the lower limbs compared to the torso, and a minimal increase in the upper and lower arms. As the air temperature increased to 37 °C, although still lower than lower limbs and torso, there was an increase in the regional sweat rates of the upper arm and lower arm. Conversely, the sweat rate on the dorsal hand tended to be higher than those on the lower limbs and torso, with a significant increase accompanying an increase in air temperature. Taylor et al. (2014) noted that the regional sweat rate on the dorsal hand was high owing to its large body surface area-to-mass ratio, which makes it suitable for heat loss. However, as shown in Fig. 1, the surface area of the dorsal hand was much lower than that of the lower limbs and torso; thus, its contribution to thermoregulation was considered to be limited.

The regional sweat rate on the palms and soles was notably higher than that in other body regions at 28 °C or lower. Additionally, under warmer environmental conditions, a proportional increase in sweat rate was not always observed with increasing air temperature. Sweating responses, characterized by features distinct from those of thermoregulatory sweating, are believed to be induced by psychological stimuli. Machado-Moreira and Taylor (2012a, 2012b) observed that the palm and sole surfaces tend to secrete more emotionally induced sweat. They demonstrated that when psychological stimuli were added during thermoregulatory sweating, the two types of sweating responses occurred simultaneously. Therefore, the regional sweat rates indicated in Fig. 2a–c for the palms and soles are believed to be influenced not only by thermoregulatory sweating but also by psychological sweating. The regional sweat rate of the forehead, as depicted in Fig. 2a and c, showed similar levels to other body regions under environmental conditions below 28 °C, which increased with rising air temperatures. However, the regional sweat rate in Fig. 2b was high under similar conditions, and no significant changes were observed with increasing air temperature. The forehead is susceptible to psychogenic sweating, similar to the palms and soles; hence, there is a possibility that it was also influenced by psychogenic sweating. Chung and Tamura (1998) reported that the differences in sweat production depicted in Fig. 2b and c were measured during midwinter and midsummer, respectively. Thus, it is possible that the seasonal acclimatization discussed in Chapter 2 influenced the results.

Figure 2d and f show the data for seated positions. In Fig. 2e and f, due to the ventral and dorsal sides of the hands and feet were not measured separately, the same values were displayed for both sides.

The data measured by the sweat pads in Fig. 2e and f were higher than those measured using the evaporimeter in Fig. 2d. This could be attributed to the sweat pad absorbing moisture from the skin surface, thus alleviating hidromeiosis and potentially increasing the local skin temperature due to contact between the sweat pad and skin surface. Assuming that it did not affect the relative relationship between the regional sweat rates of different body regions, comparisons were made.

In young, seated participants, as depicted in Fig. 2d and e, a common trend in regional sweat rates emerged. In hot environments, regional sweat rates were generally high in the torso, lower limbs, and upper limbs, excluding the hands and feet. Notably, within the torso, the sweat rate differed between the anterior and posterior surfaces, with higher regional sweat rates observed on the upper and lower back than on the chest and abdomen. While Fig. 2d, which presents the female data, shows little difference between the torso and lower limbs, Fig. 2e, which presents the male data, clearly indicates a higher sweat rate in the torso than in the lower limbs. As discussed in Chapter 2, these findings align with previous knowledge, suggesting that sweat distribution in males tends to be more concentrated in the torso compared to that in females. The regional sweat rate of older male participants depicted in Fig. 2f is similar to that depicted in Fig. 2d and e in that the posterior torso sweats more than the anterior torso; however, overall, the sweat rate was lower than that of younger male participants. In addition, unlike in Fig. 2d and e, the sweat rate of the lower limbs was lower. These characteristics align with the findings discussed in Chapter 2, indicating that aging reduces sweat production, with the decline starting in the lower limbs. As for regional sweat rates of the forehead, hands, and feet, seated position exhibited trends similar to those observed in the supine position. Nonetheless, in the supine position, regional sweat rate of the hands and feet tended to be higher than posterior torso, whereas in the seated position, an equivalent or lower tendency was observed.

Data for the standing position are shown in Fig. 2g. As mentioned earlier, the absolute values of the data were meaningless because the measurement times and skin areas were unspecified. As shown in Fig. 2g, the regional sweat rate of the torso was considerably higher than that of the limbs, with the upper limbs exhibiting a higher sweat rate than the lower limbs. The regional sweat rates of the forehead and dorsal hand were similar to those of the supine and seated positions. The regional sweat rates of the palms and soles were much lower than those in the other regions.

Based on the above, in the supine position, the regional sweat rate of the lower limbs was greater than torso; however, in the seated and standing positions, the torso’s regional sweat rate was greater than lower limbs. In all the positions, the regional sweat rate of the upper limbs was consistently lower than that of the torso. The reversal of the relative relationship between the regional sweat rates of the lower limbs and torso is believed to be influenced by the inhibitory and facilitative effects of the posture-specific sweating responses. Ferres (1960) and Tadaki et al. (1981) suggested that sweating is inhibited by the pressure on specific body regions. Kuno (1934), Kawase (1952), and Vaidya and Dhume (1994) reported that sweating is inhibited at compressed sites and promoted at opposite sites. Watkins (1956) and White et al. (1995), experimentally demonstrated that sweating promotion at opposite sites is not always guaranteed. Furthermore, Frei et al. (2019) experimentally demonstrated that sweating inhibition at a compressed site leads to an increase in body temperature, resulting in enhanced sweating at the opposite site. Therefore, the characteristics of sweat distribution between the lower limbs and torso, as indicated by the supine position data in Fig. 2a to c, and the seated position data in Fig. 2d and e, may be due to the pressure on specific body regions and thermal compensation for temperature regulation. In the supine position, the pressure primarily affects the posterior aspect of the body, particularly the back and buttocks. In the seated position, areas of the lower limbs such as the thighs and soles of the feet experience increased compression. Consequently, in the supine position, the sweating response to the torso is suppressed, likely prompting an increase in sweat production in the lower limbs as a means of coping with rising body temperature. Conversely, in the seated position, the sweating response in the lower limbs is restrained by compression, potentially stimulating sweating in the torso. In the standing position, compression is primarily exerted on the soles. This effect cannot explain the suppression of sweating across the entire lower limb.

#### Sweat distribution during exercise state

Six articles were selected for a detailed review. The experimental information collected from those articles were listed in Table 2. To facilitate comparison of the data, we organized them in a manner similar to the data obtained in the resting state. In some studies, the 13 specific regions were not measured.

**Table 2:** Experimental information from previous studies conducted under exercise state

Author	Air temp	Participants information	Exercise	Measuring method	Measurement sites (number of sites within the body part)
Weiner 1945	38 ℃	3 males (age: 31, 34, and 36 years)	Step exercise	Sealed ring	scalp, forehead, cheek, neck, shoulder (2), chest (2), abdomen (2), upper back (3), lower back, axilla (2), upper arm (2), lower arm (2), palm, dorsal hand, buttock, thigh (3), leg (3), sole, dorsal foot
Coull et al. 2021	32 ℃	14 young males (age: 24 ± 2 years), 14 old males (age: 68 ± 5 years)	Walking at 200 W.m^−2^ metabolic heat production	Sweat pad	shoulder, chest (2), abdomen (4), upper back (2), lower back (4), upper arm (2), lower arm (2), palm, dorsal hand, thigh (4), leg (3), ankle (2), sole, dorsal foot
Cotter et al. 1995	37 ℃	6 males (age: 29 ± 7 years)	Normal position cycling at 40% peak power	Ventilated capsule	forehead, chest, abdomen, upper back, lower back, upper arm, lower arm, dorsal hand, thigh, leg, dorsal foot
Patterson et al. 2000	20 ℃	10 males (age: 22 ± 5 years)	Normal position cycling at 45.5% peak power	Sweat patch	forehead, chest, abdomen, upper back, lower back, upper arm, lower arm, dorsal hand, thigh, leg, dorsal foot
Smith and Havenith 2012	26 ℃	13 females (age: 21 ± 1 years)	Running at 60% and 75% VO_2max_	Sweat pad	shoulder, chest (6), abdomen (4), upper back (2), lower back (4), upper arm (2), lower arm (2), palm, dorsal hand, thumb, finger, buttock, thigh (4), leg (3), sole, dorsal foot, ankle (2), heel, toe
Smith and Havenith 2011	26 ℃	9 males (age: 23 ± 3 years)	Running at 55% and 75% VO_2max_	Sweat pad	forehead, chin, cheek, medial head, lateral head (2), neck, shoulder, chest (2), abdomen (4), upper back (2), lower back (4), upper arm (2), lower arm (2), palm, dorsal hand, thumb, finger, buttock, thigh (4), leg (3), sole, dorsal foot, ankle (2), heel, toe

**Fig. 3:**
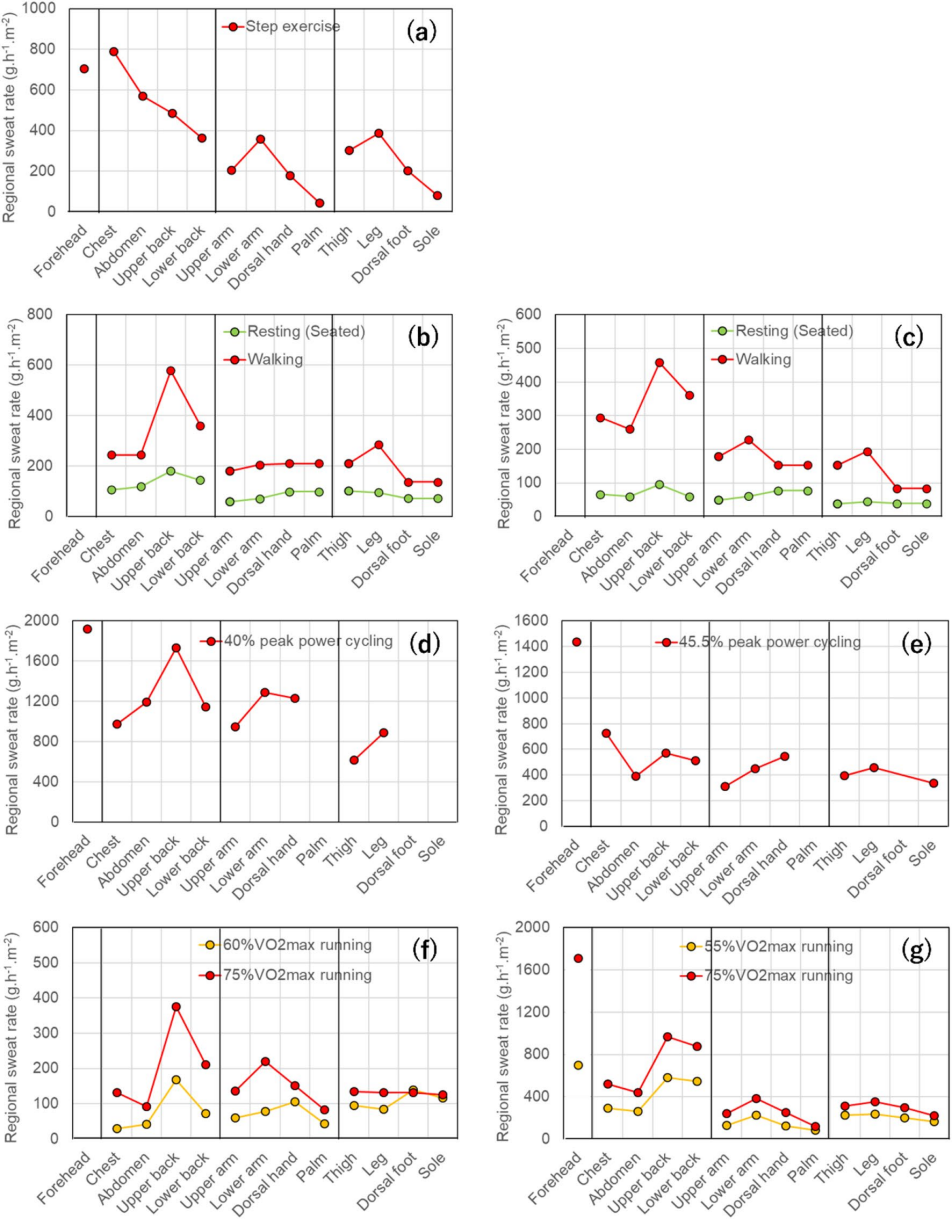
Regional sweat rate from previous studies conducted under the state of exercise. **a**: data collected by Weiner (1945), **b**: data of young participants collected by Coull et al. (2021), **c**: data of older participants collected by Coull et al. (2021), **d**: data collected by Cotter et al. (1995), **e**: data collected by Patterson et al. (2000), **f**: data collected by Smith and Havenith (2012), **g**: data collected by Smith and Havenith (2011)

Figure 3a–g present the data on regional sweat rates during the exercise state. From these figures, it is evident that the absolute values of the sweat rate varied significantly across different experiments. In particular, the data for the torso and limbs in Fig. 3d were several times larger than those for the other datasets. One factor contributing to such significant differences in absolute values could be the use of different measurement methods. Ventilated capsules (Fig. 3d), sealed rings (Fig. 3a), and sweat pads (Fig. 3b, c, e-g) were used. As mentioned earlier, ventilated capsules tend to enhance sweat secretion; thus, the high sweat rate indicated in Fig. 3d was largely influenced. Furthermore, with regard to the experimental data other than Fig. 3d, it is considered that they were not collected from the skin surface in its natural state, indicating the prevalence of differences. Assuming that the relative relationship of the regional sweat rates among different body regions did not significantly deviate from their natural state, we compared the data accordingly.

In addition to the measurement methods, the difference between the metabolic rate and absolute external work rate (metabolic heat production) of the participants significantly influenced sweat rate. However, metabolic heat production was not reported in those studies except for Coull et al. (2021). Therefore, based on the information regarding the participants’ characteristics and exercise intensity provided in each study, metabolic heat production was calculated as shown in Table 3. The calculation process is detailed in the Appendix 2.

**Table 3:** Estimation of metabolic heat production during exercise state

Author	Estimated metabolic heat production
Weiner (1945)	98–112 W.m^−2^
Coull et al. (2021)	200 W.m^−2^
Cotter et al. (1995)	270 W.m^−2^
Patterson et al. (2000)	270 W.m^−2^
Smith & Havenith (2012)	425 W.m^−2^ for 60% VO_2 max_ and 550 W.m^−2^ for 75% VO_2 max_
Smith & Havenith (2011)	500 W.m^−2^ for 55% VO_2 max_ and 700 W.m^−2^ for 75% VO_2 max_

Sweat data of young individuals were examined from the perspective of metabolic heat production. As shown in Fig. 3a, when the metabolic heat production was minimal, the anterior torso showed a slightly higher regional sweat rate than the limbs. Next, as shown in Fig. 3b, where the metabolic heat production was 200 W.m^−2^, only the posterior torso exhibited a higher sweat rate, while differences among other body regions were minimal. As shown in Fig. 3d–g, where the metabolic heat production was high, there was a tendency for a higher sweat rate in the torso. Moreover, as shown in Fig. 3f and g, under conditions of greater metabolic heat production, the regional sweat rate was generally higher across all regions, with the most notable difference observed in the posterior torso. The regional sweat rate of the forehead was measured in four experiments, all of which showed consistently high values. Conversely, the hands and feet had moderate to below average rates compared with all other body regions in each experiment, accordingly, comparatively lower thermoregulatory contribution.

Based on the above, sweat distribution during exercise state tends to favor the torso. Additionally, with increasing metabolic heat production, there is a possibility of increased sweat distribution to the forehead, and the relative distribution among other body regions does not change significantly. Upon reviewing these figures, it became evident that in many exercise conditions, sweat production tends to be greater on the back, followed by the front of the torso, and then the limbs. Nevertheless, Fig. 3a and e show that the front of the torso exhibits a higher sweat production tendency. Sweat pads were used in the experiment, as shown in Fig. 3e. The surface area of sweat pads applied to areas such as the chest and back was only 24 cm^2^, which may have resulted in inaccurate measurements of regional sweat rates. In the experiment illustrated in Fig. 3a, the experimental conditions were not adequately controlled. Considering that they were based on older measurements, there may have been accuracy issues with the regional sweat rates. Regarding the characteristic of greater sweating on the back, Havenith et al. (2008) suggested that it might be a remnant of the time humans walked on all fours before evolution, where the back, exposed more extensively to the external environment, efficiently facilitated sweat evaporation compared to the front.

Among the six studies, Fig. 3f and g share consistent experimental settings, including environmental conditions, exercise protocols, and measurement methods. Comparing the data of female at 75% maximal oxygen consumption from Fig. 3f with those of male at 55% maximal oxygen consumption (Fig. 3g), it was evident that male exhibited higher sweat rates than female. A pronounced sex difference became apparent, particularly when metabolic heat production was high, which is consistent with the findings reviewed in Chapter 2. Figure 3b and c depict the experiments conducted with both young and older participants. In older individuals, sweat distribution followed a pattern similar to that of young individuals, with a tendency for a greater sweat rates in the posterior torso, followed by the anterior torso and limbs, albeit generally lower. Thus, a decline in sweat rate due to aging was observed not only in the resting state but also during the exercise state. When comparing Fig. 3d and e, which depict cycling while seated, with Fig. 3a to c, f, and g, which illustrate step exercise, running, and walking while standing, respectively, no clear differences in sweat distribution were observed due to posture. One possible reason might be that both postures involve minimal differences in the upright position.

#### Local effect

It has been argued that parameters, including not only body temperature and average skin temperature, but also local skin temperature, contribute to the local sweating response (van Beaumont and Bullard 1965; Bullard et al. 1970). Experimental evidence has demonstrated that local skin temperature modulates local sweating responses by regulating sweat rate and the threshold of sweating onset (Nadel et al. 1971a, 1971b; Elizondo and Bullard 1971). The local effects of skin temperature are generally expressed by the temperature coefficient Q_10_: Q_10_ represents the sensitivity of a biological process to a 10 °C temperature change, indicating how much the process varies with temperature fluctuations. The definition of Q_10_ is as follows:

3 $$\frac{{R}_{2}}{{R}_{1}}={{Q}_{10}}^{\frac{{T}_{2}-{T}_{1}}{10}}$$

where T is the temperature (in degrees Celsius or Kelvin); R is the reaction rate; and R_1_ and R_2_ are the reaction rates measured at T_1_ and T_2_, respectively.

Table 4 summarizes the values of the temperature coefficient Q_10_ as reported in previous studies. Bullard et al. (1967) reported that they varied the skin temperature of the participants’ 10-cm^2^ thigh area under five environmental conditions of air temperature ranging from 31 to 39 °C. They measured the regional sweat rate of the thigh and found that Q_10_ was 2–3 when the air temperature was below 33 °C and 4–5 when it exceeded 33 °C. Nadel et al. (1971a) controlled the average skin temperature using radiant heat exposure and heated or cooled the skin temperature of a 12-cm^2^ area on the thigh using capsules with warm or cold water perfusion. Based on the regional sweat rate of the thigh during this process, the authors reported a Q_10_ value of approximately 3. Ogawa and Asayama (1986) reported a Q_10_ of 2.52 based on the regional sweat rate of the forearm when subjected to heat irradiation.

**Table 4:** Summary of local effect

Author	Air temp	Changes in local skin temperature	Measurement site	Q_10_ coefficient
Bullard et al. 1967	< 33 ℃	38–44 ℃	Thigh	2–3
	> 33 ℃	21–43 ℃	Thigh	4–5
Nadel et al. 1971a	25–35 ℃	30–38 ℃	Thigh	3
Ogawa and Asayama 1986	35–41 ℃	37–42 ℃	Forearm	2.52

Regarding the mechanism of local temperature effects, MacIntyre et al. (1968) experimentally demonstrated that local skin heating increases the release of acetylcholine, a neurotransmitter, from the neuroglandular junction. Elizondo (1973) made a similar statement. In contrast, Ogawa (1970) suggested that an increase in local skin temperature not only increases the amount of neurotransmitters released but also enhances the sensitivity of sweat glands.

#### Thermosensitivity

Peripheral thermoreceptors that receive thermal stimuli from the external environment transmit the resulting neural signals to the thermoregulatory centers. If sensory responses to thermal stimuli vary among different body regions, the generated neural signals will also differ. Previous studies on thermosensitivity have predominantly investigated the subjective sensations of the participants, revealing that the thermosensitivity of the forehead is the highest across the body (Cotter et al. 1996; Shimazaki et al. 2012; Gerrett et al. 2014; Inoue et al. 2016). Moreover, it has been demonstrated that individuals are more sensitive to cold stimuli than to heat stimuli, indicating higher thermosensitivity to cold (Gerrett et al. 2015; Schmidt et al. 2020). Although distinct thermosensitivity may not only reflect subjective sensations to thermal stimuli, but also contribute to thermoregulation responses, research in this field is limited. Table 5 summarizes thermosensitivity results reported in previous studies. The values presented here were extracted from the data in the figures and tables of each study and normalized to establish an average value of 1 across the entire dataset.

**Table 5:** Summary of thermosensitivity coefficient

	Nadel et al. 1973 (*N* = 2)	Crawshaw et al. 1975 (*N* = 5)	Patterson et al. 1998 (*N* = 8)		Cotter and Taylor 2005 (*N* = 13)		
	Warm stimulus	Cool stimulus	Warm stimulus of 3 ℃	Cool stimulus of 3 ℃	Warm stimulus of 4 ℃	Cool stimulus of 4 ℃	Cool stimulus of 11 ℃
Face	2.6	2.4	2.1	1.5	1.8**	3.9*	2.1**
Chest	1.0	0.8			1.0	0.1	1.0
Abdomen	0.8	0.6			1.7	0.4	1.0
Back	1.1	0.9			1.4	0.8	1.5
Upper arm	0.8	0.9	0.7		0.9	0.1	1.1
Forearm	0.4	0.9	0.1	0.3	0.4	0.4	0.3
Hand			1.1	1.2	0.4	0.2	1.0
Thigh	0.8	0.7			1.1	0.9	0.6
Leg	0.4	0.4			1.0	1.6	0.7
Foot					0.3	1.7	0.7
** *P* < 0.01, * *P* < 0.05: significant difference from body region							

Nadel et al. (1973) quantified thermosensitivity by locally heating several body regions in a 36 °C environment and measuring the regional sweat rate of the thigh. They found that the thermosensitivity of the forehead was three times higher than that of the thigh, the chest and abdomen exhibited thermosensitivities similar to that of the thigh. Thermosensitivity in the distal limbs, namely, the lower leg and forearm, was approximately half of that in the thigh. In the experiment conducted by Crawshaw et al. (1975), participants were exposed to a hot environment ranging from 39 to 40 °C while cooling stimuli was applied to parts of their bodies, altering the local skin temperature from 36 °C to 16–22 °C and measuring the reduction in thigh’s regional sweat rate. They quantified thermosensitivity to cool stimuli across different body regions and found that the thermosensitivity of the forehead was approximately three times higher than that of the thigh but reported no significant differences in sensitivity among other body regions. Patterson et al. (1998) pointed out several issues with these previous studies, including the lack of control over the participants’ bodies and average skin temperatures, discrepancies between the stimulated skin area and skin temperature changes, and the absence of statistical analysis. Patterson et al. addressed these issues and investigated the effects of local heating and cooling (± 3 °C) on the forehead, upper arm, forearm, and hand of the upper body. Although differences in thermosensitivity were observed between body regions, no statistically significant differences were found. Cotter and Taylor (2005) further refined Patterson’s experiment by introducing three types of stimuli, 4 °C heating, 4 °C cooling, and 11 °C cooling, and expanded the measurement sites from four upper body regions to ten throughout the entire body. With the 4 °C heating stimulus, the thermosensitivity of the forehead was significantly higher than that of the upper and lower limbs as well as limb extremities (*P* < 0.01). In the case of 4 °C cooling stimulus, thermosensitivity of forehead was significantly higher than that of the upper limbs (*P* < 0.05). With the 11 °C cooling stimulus, the thermosensitivity of the forehead significantly surpassed that of all other body regions (*P* < 0.01). Moreover, the thermosensitivity of the torso to heating stimuli was second only to that of the forehead, whereas it decreased towards the limbs, particularly the extremities. In cooling stimuli, thermosensitivity across body regions was inconsistent between the 4 °C and 11 °C conditions, with no discernible pattern. Collectively, these four experiments demonstrated that the forehead consistently exhibits higher thermosensitivity.

## Discussion

This study conducted a literature review of the individual characteristics and local body functions that influence the sweating response. We organized insights into individual characteristics, including aerobic fitness, age, heat acclimation, sex, and body surface area-to-mass ratio. Subsequently, we selected previous studies on local body functions, such as sweat distribution, local effects, and thermosensitivity, and compared the experimental data.

The impact of individual characteristics is as follows: promoting the sweating response involved enhancing aerobic fitness, artificial heat acclimation over several weeks, and seasonal heat acclimatization over several months. Inhibition of the sweating response is attributed to aging and geographic heat acclimatization, which develops over extended periods. Sex differences were not apparent when metabolic heat production was low; as metabolic heat production increased, male tended to sweat more than female. The body surface area-to-mass ratio is significantly related to the balance between sensible and latent heat loss through sweating. Individuals with a smaller body surface area-to-mass ratio (i.e., larger body size) rely more on heat loss through sweating for thermoregulation. As mentioned above, much has been elucidated regarding the influence of individual characteristics on the sweating response.

In terms of local body function, sweat distribution indicates that in hot environments during the resting state, the increase is particularly significant in the lower limbs and torso. Furthermore, the regional sweat rate of the lower limbs is higher in the supine position, whereas the torso exhibits a higher sweat rate in the seated position. It was inferred that increasing sweating in the lower limbs provides two benefits: First, the relatively large surface area and high evaporative heat transfer coefficient allow for greater heat dissipation. Second, due to the long blood flow pathways in the lower limbs, cooling these parts can efficiently cool the blood. By prioritizing the reduction in skin temperature in the lower limbs through sweating and circulation of a large volume of blood, it is possible to lower blood temperature efficiently, thereby preventing an increase in body temperature. Regarding the increase in sweating on the torso, it is believed that compared to the thighs, the blood flow pathways in the torso are shorter, and the evaporative heat transfer coefficient is not as high. Therefore, the efficiency of lowering the blood temperature is considered to be much lower than that of the lower limbs. Nevertheless, the surface area of the torso comprises a significantly large proportion of the total body surface area (Fig. 1). Reducing the skin temperature of the torso through sweating can lead to a significant increase in heat conduction from the core to the body surface. Regarding the difference in sweat distribution due to posture, it is considered that in the supine position, sweating is mainly suppressed in the back and buttocks due to compression, while sweating in the lower limbs increases with thermoregulatory compensation. Similarly, in the seated position, sweating suppression is primarily observed in the thighs and soles due to compression, whereas sweating in the torso region is presumed to be stimulated. Explaining sweat distribution in the standing position proved difficult by compression. Notably, only one experiment was conducted to measure sweating in the standing position, raising concerns regarding the reliability of the collected data.

Regarding sweat distribution during exercise, regardless of metabolic heat production, there is a higher sweat output in the upper and lower back, whereas the regional sweat rate in the limbs is relatively lower. A substantial amount of metabolic heat is generated during exercise. However, when attempting to dissipate this heat through the limbs, as in the resting state, the available surface area for latent heat loss is restricted. In addition, increased blood circulation to the limbs imposes strain on the heart. Moreover, maintaining blood flow from the heart to the muscles during exercise necessitates a certain amount of blood circulation, further burdening the heart if blood flow to the skin is increased. Therefore, it is inferred that distributing a significant amount of sweat in the torso to lower the skin temperature and increase heat conduction from the core to the surface reduces the burden on the body. Differences in sweat distribution were observed between the resting and exercise states. In accordance with each condition, the sweat rate of different body regions is reasonably controlled to maintain the body temperature.

The existence of local effects was confirmed by multiple experiments that revealed temperature coefficients ranging from 2 to 5. Previous studies have only elucidated local effects on the thigh and forearm, leaving uncertainty regarding whether similar Q_10_ values exist in other body regions. Regarding thermosensitivity, multiple experiments have demonstrated that among all body regions, only the forehead exhibits a significantly high level. However, experimental data are limited, leaving room for further exploration of the differences among regions other than the forehead. Further research is required to provide more detailed insights into local effects and thermosensitivity.

Although studies investigating the individual characteristics influencing sweating response have been extensively conducted, only few studies have comprehensively reviewed these aspects. In particular, few literature reviews have encompassed recent research findings. Baker (2019) focused on the relationship between sweat components and human health, centralizing the review of the impact of electrolytes contained in sweat. Cramer et al. (2022) focused primarily on how individual characteristics influence body temperature through sweating response, thus providing a limited reference for the sweating response itself. In contrast, this study not only reviewed the impact of individual characteristics, but also the mechanisms underlying sweating promotion and inhibition.

Several review articles have addressed sweat distribution and the local body functions; yet there is a scarcity of studies that summarize past experimental data, examining both their commonalities and differences. Most of these studies focused only on limited regions of the body, leaving the overall sweat distribution across the body unexplored (Arens and Zhang 2006; Smith and Johnson 2016). In contrast, Taylor and Machado-Moreira (2013) are the only authors to conduct a comprehensive review to date. However, they did not consider the impact of differences in measuring methods and experimental conditions across various studies. They calculated simple averages of all experimental data without considering these differences. This study demonstrated significant variations in sweat rate depending on the measurement method, and conducted an analysis focusing on the relative relationships between body regions. Furthermore, we categorized the data based on experimental conditions and revealed that the features of sweat distribution vary according to these conditions. Regarding local effects and thermosensitivity, detailed review surveys addressing these aspects are lacking. This study represents the first comprehensive review survey conducted on these topics, in which the experimental conditions and quantitative results from previous studies are systematically organized.

The accumulation and achievements of previous studies on the influence of individual characteristics and local body functions on sweating responses have been demonstrated in this study. These results are expected to aid the analysis and interpretation of future thermophysiological experiments and contribute to the understanding of the human thermoregulatory system.

## Conclusion

Individual characteristics that affect the sweating response include enhanced aerobic fitness and aging, each exerting a stimulating and inhibitory effect. The effect of heat acclimation on sweating varies depending on the duration of the acclimation period. Although several weeks of artificial heat acclimation and several months of seasonal heat acclimatization enhanced the sweating response, geographic heat acclimatization spanning several years had a suppressive effect. When the metabolic heat production is high, male tend to sweat more than female. Individuals with smaller body surface area-to-mass ratios rely more on sweating responses for thermoregulation. In terms of sweat distribution in the resting state, the regional sweat rate was higher in the lower limbs and torso, with the lower limbs exhibiting a greater sweat rate than the torso in the supine position, and the torso having a greater sweat rate than the lower limbs when seated. During exercise, the regional sweat rate in the torso was high, but those in the limbs were relatively low. Such a distribution is inferred to efficiently regulate body temperature while minimizing the physiological strain due to heat stress. Local effects were examined only on the thighs and forearms, with temperature coefficients Q_10_ ranging from 2 to 5. Regarding thermosensitivity, only the forehead showed significantly higher values among all the body regions.

## Supplementary Information

Below is the link to the electronic supplementary material.

Supplementary file1 (DOCX 374 KB)

## Data Availability

Data will be made available on request.
