# Supplementary material for: Effects of individual characteristics and local body functions on sweating response: A review
Source: Int J Biometeorol. 2024 Aug 14;68(11):2185–204. doi: 10.1007/s00484-024-02758-7 (PMC11519300; doi:10.1007/s00484-024-02758-7)
Supplement: Supplementary file 1 — Supplementary file1 (DOCX 374 KB) [file 484_2024_2758_MOESM1_ESM.docx]

# Appendix 1

A literature search was conducted on the PubMed, Scopus, Web of Science, and J-STAGE databases. The keywords included in the search terms in Chapter 2, which are related to sweating response and various individual characteristics, are summarized in Table A1. Flow charts of the literature search for Chapter 3 on (1) sweat distribution, (2) local effect and (3) thermosensitivity are summarized in Fig. A1, A2 and A3, respectively.

| **Table A1 Search terms used for literature search described in Chapter 2** | |
| --- | --- |
| Topic | Search terms |
| Sweating response | Sweat, sweating, sweating response, sweat rate, sweat production |
| Individual characteristics | Age, aging, old, elderly |
|  | Acclimation, acclimatization, adaption, tolerance |
|  | Sex, gender, female, women |
|  | Body surface to mass ratio, body surface area, body size, morphology |
|  | Aerobic fitness, physical training, maximum oxygen consumption, endurance training, exercise |

| 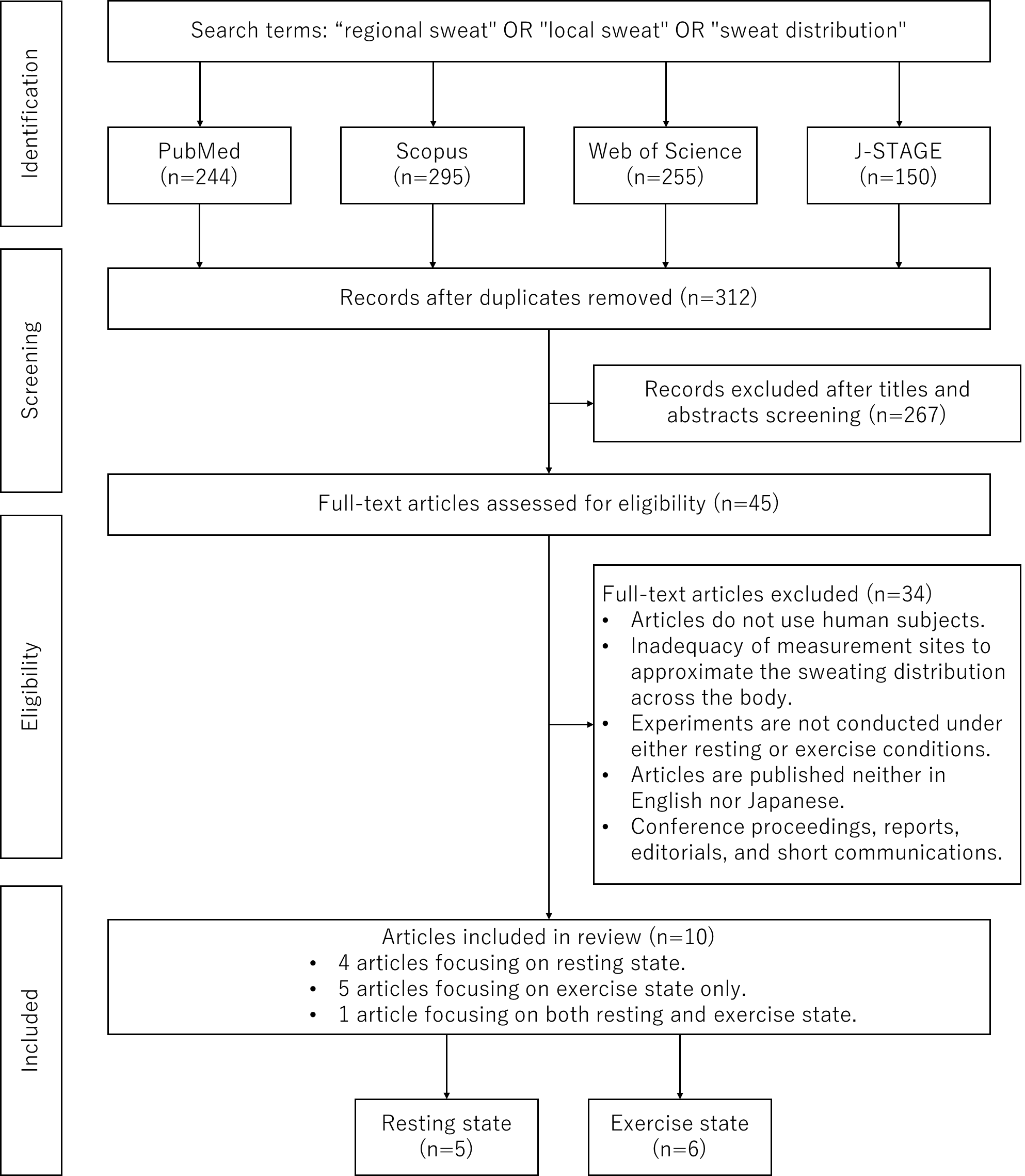 |
| --- |
| **Fig. A1 Flow chart of literature search on sweat distribution** |

| 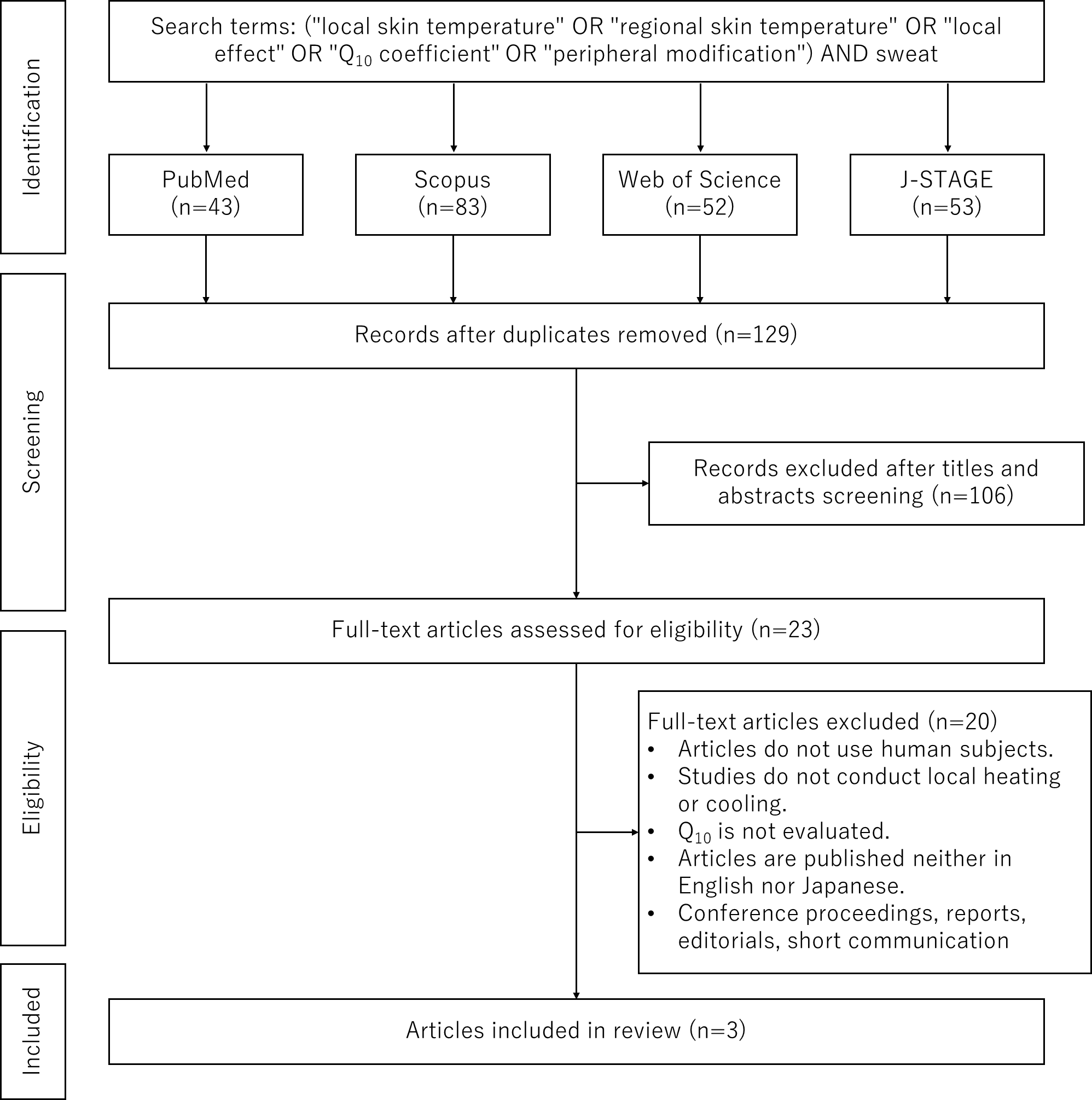 |
| --- |
| **Fig. A2 Flow chart of literature search on local effect** |

| 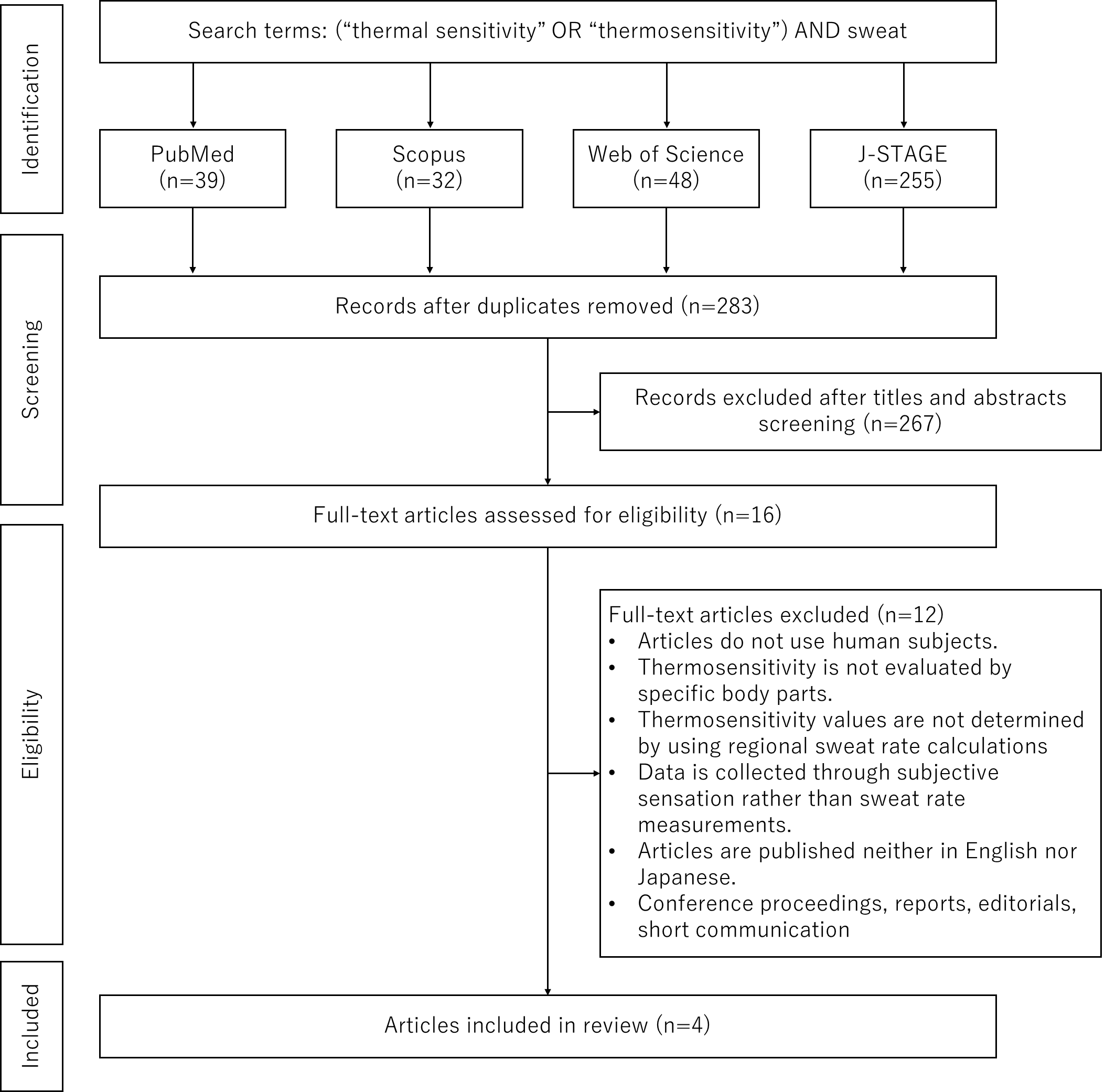 |
| --- |
| **Fig. A3 Flow chart of literature search on thermosensitivity** |

# Appendix 2

To analyze sweat distribution during exercise, we calculated the metabolic rate and metabolic heat production based on the information provided in each study.

The calculation of metabolic rate [W.m^-2^] employed the following equation (Nishi, 1981), except for Weiner (1945):

| $M=\frac{\dot{V}_{O_{2}}}{60}\times\frac{e_{c}\left( R-R_{f} \right)+e_{f}\left( R_{c}-R \right)}{R_{c}-R_{f}}\times\frac{1}{BSA}$ | (A1) |
| --- | --- |

Where $\dot{V}_{O_{2}}$ is oxygen consumption [L.min^-1^]; R is respiratory quotient; R_c_ is the respiratory quotient when consuming carbohydrates only (=1.0); R_f_ is the respiratory quotient when consuming fats only (=0.7); e_c_ is the heat production per liter of oxygen consumed during carbohydrate oxidation (= 21,130) [J.L^-1^]; e_f_ is the heat production per liter of oxygen consumed during fat oxidation (= 19,690) [J.L^-1^]; and BSA is body surface area [m^2^].

The values of the respiratory quotient R used in this study are presented in Table A1. The respiratory quotient concerning Cotter et al. (1995) and Patterson et al. (2000) was set based on Parsons (2003). Smith and Havenith (2011, 2012) set the respiratory quotient values in their study.

| Table A1 Respiratory quotient ratio of exercise conditions | |
| --- | --- |
| Author | Respiratory quotient |
| Cotter et al. (1995) | 0.85 |
| Patterson et al. (2000) | 0.85 |
| Smith & Havenith (2012) | 0.85 for 60%VO_2_ _max_ and 1 for 75%VO_2_ _max_ |
| Smith & Havenith (2011) | 0.85 for 55%VO_2_ _max_ and 1 for 75%VO_2_ _max_ |

The metabolic heat production H_prod_ [W_._m^-2^] of the participants is as follows:

| $H_{prod}=M-W$ | (A2) |
| --- | --- |

where W is the absolute external work rate [W.m^-2^].

Cotter et al. (1995) and Patterson et al. (2000) provided peak power instead of VO_2_ _max_. Therefore, the following equation (Hawley and Noakes 1992) was used to calculate VO_2_ _max_ [L.min^-1^]:

| $\dot{V}_{O_{2}max}=0.01141\times W_{peak}+0.435$ | (A3) |
| --- | --- |

where W_peak_ is the peak power [W].

Additionally, because Patterson et al. (2000) did not provide participant height data, a height of 180 cm was assumed, and the body surface area [m^2^] was calculated using the DuBois formula (DuBois and DuBois 1916):

| $BSA=0.007184\times{Weight}^{0.425}\times{Height}^{0.725}$ | (A4) |
| --- | --- |

where Weight and Height are the participant’s weight [kg] and height [cm], respectively.

Weiner (1945) provided no information on exercise intensity. Therefore, considering the heat generated by muscles during the step exercise against gravity, the metabolic heat production of the participants was calculated using the following formula (Tanabe et al. 1995):

| $H_{prod}=\frac{Weight\times g\times h\times N_{step}}{\eta\times T\times BSA}+M_{rest}$ | (A5) |
| --- | --- |

Where g represents the gravity acceleration [m.s^-2^]; h is the vertical distance moved per step (= the height of the step) [m.step^-1^]; N_step_ is the total number of steps ascended during the exercise period [step]; η is muscle work efficiency (= 0.15); T is the total time of the exercise and non-exercise periods [s]; and M_rest_ is the metabolic rate under non-exercise conditions (assumed to be 58.15 W.m^-2^ in this study).
